# Satisfactory mid-term outcomes of condylar-constrained knee implants in primary total knee arthroplasty: clinical and radiological follow-up

**DOI:** 10.1186/s10195-020-00561-9

**Published:** 2020-12-02

**Authors:** Fabio Mancino, Ivan De Martino, Aaron Burrofato, Carmine De Ieso, Maristella F. Saccomanno, Giulio Maccauro, Vincenzo De Santis

**Affiliations:** 1 Division of Orthopaedics and Traumatology, Department of Aging, Neurological, Orthopaedic and Head-Neck Studies, Fondazione Policlinico Universitario Agostino Gemelli IRCCS, 00168 Rome, Italy; 2 Università Cattolica del Sacro Cuore, Largo Francesco Vito 1, 00168 Rome, Italy; 3 Department of Orthopaedics, Children’s Hospital “Bambino Gesù”, Rome, Italy; 4 Villa Stuart Sport Clinic, FIFA Medical Centre of Excellence, Rome, Italy; 5 Department of Orthopaedics and Traumatology, Mater Olbia Hospital, Olbia, Italy

**Keywords:** Constrained condylar knee, Varus–valgus constraint, Total knee arthroplasty, Primary TKA, Valgus deformity, Coronal deformity, Survivorship, Instability, Joint laxity, CCK

## Abstract

**Background:**

The purpose of this study was to evaluate (1) the reoperation rates and survivorship for septic and aseptic causes, (2) radiographic outcomes, and (3) clinical outcomes of condylar-constrained knee (CCK) implants used in primary total knee arthroplasty (TKA) with severe coronal deformity and/or intraoperative instability.

**Materials and methods:**

A consecutive series of CCK implants in primary TKA was retrospectively evaluated in patients with severe coronal deformities. Forty-nine patients (54 knees) were included with a mean follow-up of 9 years (range 6–12). All patients were treated with a single-design, second-generation CCK implant. The primary diagnosis was osteoarthritis in 36 knees, post-traumatic arthritis in 7 knees, and rheumatoid arthritis in 4 knees. Preoperatively, standing femorotibial alignment was varus in 22 knees and valgus in 20 knees.

**Results:**

At a mean follow-up of 9 years, overall survivorship was 93.6%. Two knees (4.3%) required revision for periprosthetic joint infection. One knee (2.1%) required subsequent arthroscopy due to patellar clunk syndrome. At final follow-up, no evidence of loosening or migration of any implant was reported, and the mean Knee Society knee scores improved from 43 to 86 points (*p* < 0.001). The mean Knee Society function scores improved to 59 points (*p *< 0.001). The average flexion contracture improved from 7° preoperatively to 2° postoperatively and the average flexion from 98° to 110°. No knees reported varus–valgus instability in flexion or extension.

**Conclusion:**

CCK implants in primary TKA with major coronal deformities and/or intraoperative instability provide good midterm survivorship, comparable with less constrained implants. In specific cases, CCK implants can be considered a viable option with good clinical and radiographic outcomes. However, a higher degree of constraint should be used cautiously, leaving the first choice to less constrained implants.

**Level of evidence** Therapeutic study, level IV.

## Introduction

Total knee arthroplasty (TKA) is one of the most frequent orthopedic surgical procedures, with > 600,000 surgeries performed every year in the United States and the number is projected to grow by 673% in 2030 [1]. Despite the excellent reported results, with registries reporting survivorship of > 95% after 10 years [2, 3], aseptic loosening and instability are among the most common failure mechanisms requiring revision TKA [4–6].

In the majority of primary TKAs, a well-balanced and stable knee is achieved through soft-tissue balancing, and a variable constraint provided by a standard cruciate-retaining (CR), posterior-stabilized (PS) or medial-stabilized (MS) implant. However, in severe coronal deformities with stretched or incompetent collateral ligaments or when intraoperative balancing cannot be adequately achieved with standard implants, a higher degree of constraint may be required [7–9]. Condylar-constrained knee (CCK) prostheses are characterized by a tibial post higher and more squared than the PS one, that fits intimately between the condyles and provides medial–lateral and anteroposterior (AP) stability limiting varus–valgus and torsional moments [10, 11]. First-generation CCK implants were non-modular and were associated with a high rate of aseptic loosening [7] and a high rate of patellar complications including fracture, maltracking and osteonecrosis [12]. Second-generation CCK implants, with a redesigned patellofemoral articulation, provide increased medial–lateral stability and allow modular tibial and femoral stems in order to enhance fixation, provide stress relief and load-sharing within the host bone [13].

Theoretical disadvantages have been addressed to an increased level of constraint including greater risk of loosening caused by the transmission of the mechanical stress to the bone–cement interface and subsequent increased polyethylene insert wear, particularly of the tibial post, leading to a higher prevalence of osteolysis [11, 12, 14–17]. However, multiple studies have reported good short-to-midterm results of CCK implants in primary TKAs. In addition, range-of-motion (ROM) [17], clinical and radiographic outcomes seem to be comparable to standard PS implants in severe coronal deformity and ligamentous laxity [11, 12, 15, 18–23].

Therefore, the purpose of this study was to evaluate (1) the reoperation rates and survivorship for septic and aseptic causes, (2) radiographic outcomes, and (3) clinical outcomes based on Knee Society scores of a single-design second-generation CCK implant used in primary TKAs with severe coronal deformity and/or medial–lateral instability.

## Patients and methods

We retrospectively evaluated the records of 49 consecutive patients (54 knees) who underwent primary TKA using a single-design, second-generation condylar-constrained implant (NexGen LCCK; Zimmer, Warsaw, IN, USA) at one single institution between 2005 and 2012. The patients were identified from our institutional joint replacement database, ensuring a minimum follow-up of 5 years. We included all patients treated with primary knee arthroplasty in which the NexGen LCCK was implanted. During this period, 1,006 primary TKAs were performed at the same institution. Four patients (8.2%) were lost during follow-up and three patients (6.1%) died for reasons not related to index surgery, leaving 42 patients (47 knees) for final evaluation. The decision to use a CCK implant was left to the surgeon to decide depending on the patient’s deformity, preoperative ligamentous stability, and intraoperative assessment of competency of the collateral ligaments and coronal stability after soft-tissue release was performed. In 12 knees (out of 47, 26%), the decision to use a CCK implant was taken preoperatively in either a valgus deformity ≥  20° (4 of 47, 9%; mean 22.5°, range 20–25°) or a varus deformity ≥ 20° (8 of 47, 17%; mean 23.8°, range 20–30°) with or without collateral ligament failure. In the rest of the knees the decision was taken intraoperatively after attempting to balance the knees with a PS prosthesis; inadvertent intraoperative sectioning of the medial collateral ligament was reported in 2 knees (4.3%). Preoperatively, standing femorotibial alignment was varus in 22 knees (46.8%) averaging 13.3° (range 5–30°). Valgus deformity was present in 20 knees (42.6%) averaging 14.2° (range 5–25°). Overall, 42 knees (89.4%) had > 5° coronal deformity, including 17 knees (36.2%) with major coronal deformity > 15°.The remaining 5 knees (10.6%) were neutral or had little deformity. All patients were followed clinically and radiographically for a mean of 9 years (range 6–12 years). The cohort included 34 women (81%) and 8 men with an average age of 72 years (range 43–86 years) at the time of index surgery. Mean body mass index was 29 kg/cm^2^ (range 18–37 kg/cm^2^). There were 26 right (55%) and 21 left knees (45%). The underlying diagnosis that led to the initial primary TKA was osteoarthritis in 31 patients (36 knees, 76%), post-traumatic arthritis in 7 patients (7 knees, 15%), and rheumatoid arthritis in 4 patients (4 knees, 9%) (Table 1).

**Table 1:** Demographic details of the 42 patients (47 knees) in the final cohort

Variable	Value
Number of knees	47
Number of patients	42
Gender	
Female	34 (81%)
Male	8 (19%)
Side (number)	
Right	26 (55%)
Left	21 (45%)
Diagnosis (number of knees)	
Osteoarthritis	36 (76%)
Post-traumatic osteoarthritis	7 (9%)
Rheumatoid arthritis	4 (15%)
Body mass index (kg/m^2^) (range)	29 (18–37)
Mean age at surgery (range; years)	72 (43–86)
Varus deformity (°) (range; mean)	
5–30° (13.3°)	22 (46.8%)
20–30°	8 (17%)
Valgus deformity (°) (range; mean)	
5–25° (14.2°)	20 (42.6%)
20–25°	4 (8.5%)

The surgical technique for the implantation of a CCK implant has already been described [14, 19]. All the surgeries were performed by 2 experienced knee surgeons with preference for PS implants. The surgeries were performed under general or regional anesthesia. Tourniquet and a standard medial parapatellar approach via a midline skin incision were used in all cases. The distal femoral cut was performed using an intramedullary guide and the femoral rotation was set in neutral rotation relative to the transepicondylar axis. The tibial resection was aligned with an extramedullary guide perpendicular to the long axis of the tibia according to the surgical technique. The varus–valgus stability was evaluated at 0° extension, mid-flexion (30–40°) and 90° flexion with a spacer block or a trial PS component. If the knee was considered unstable with a varus or valgus laxity of > 3 mm at any point of the ROM, after a concerted effort to obtain balance, a condylar-constrained implant (LCCK®; Zimmer) was used. Stem extensions were used in all cases (100%). Short straight femoral stems sizing 15 × 30 mm were used in 44 out of 47 knees (93.6%), with 18 × 100 mm stems being used in 2 knees (4.3%) and 20 × 100 mm in 1 knee (2.1%). On the tibial side, a short straight 15 × 30 stem was used in 44 knees (93.6%) and 14 × 100 mm in 3 knees (6.4%)—2 of them were straight and 1 with offset. Short stems (30 mm) were fully cemented and cement was placed around the components and digitally impacted into the femoral and tibial metaphysis. Long stems (100 mm) were fixated with the hybrid technique. Eventual tibial or femoral bone defects were carefully assessed intraoperatively using the Anderson Orthopaedic Research Institute (AORI) Classification System [24] and rated as Type 2A in four tibias (4/47, 8.5%). In those cases, a finned precoat half block tibial augment (Zimmer) was used, sizing 5 mm in three cases and 10 mm in one case. Bone grafts were not used in any knee. Nine patellae (of 47, 19.1%) were resurfaced using a 3-peg all-polyethylene component with a reported mean size of 32 mm. In the remaining 38 knees, the patella was left unresurfaced because of good bone quality. Patellar thermal denervation with electrocautery was performed in all cases. Antibiotic-loaded bone cement Simplex™ P with Tobramycin Bone Cement (Stryker®, Mahwah, NJ, USA) was used routinely in all patients. An intra-articular closed suction drain was used at the end of every surgical procedure and removed within 24–48 h postoperatively. Each patient received intravenous perioperative antibiotic prophylaxis, multimodal pain management and routine postoperative prophylaxis for thromboembolic disease with low-molecular-weight heparin. All patients were permitted weight-bearing as tolerated using crutches or a cane as necessary, beginning on the first postoperative day. The rehabilitation protocol with active ROM exercises and progressive weight-bearing was reached.

Patients were followed clinically and radiographically at the preoperative visit, 4 weeks postoperatively, and at 3, 6, and 12 months and then yearly thereafter. All patients were seen within 12 months of data collection for this study. Knee function was assessed with the Knee Society scoring system [25]. Radiographic review was performed by an independent fellowship-trained orthopedic surgeon according to the Knee Society TKA radiographic evaluation system [26] modified for long-stemmed revision prostheses [27], updated by Meneghini et al. [28]. A plain AP radiograph and a lateral knee plain radiograph were performed prior to discharge. Radiographs, consisting of standing AP, lateral, and Merchant views, were evaluated for any sign of loosening, including subsidence, cement mantle fractures, radiolucent zones, radiolucent lines, and implant migration. Fluoroscopic views were not used, but a standardized protocol with experienced radiology technicians was used.

Continuous variables were described using means and ranges. Categorical variables were described using absolute frequencies. To analyze differences in functional score (Knee Society score) collected before surgery and those collected at final follow-up, a two-tailed paired *t*-test and Wilcoxon signed-rank test were used. All statistical analyses were performed using SPSS v.26 (SPSS Chicago, IL, USA). *P* values of < 0.05 were considered significant.

Institutional review board approval was obtained before initiation of this study from the Catholic University of the Sacred Heart Research Ethics Board, Number 13397/14.

## Results

At a mean follow-up of 9 years, survivorship with reoperation for any reason as an end-point was 93.6%. With implant revision for aseptic loosening or osteolysis as end-point, survivorship was 100%. With implant revision for periprosthetic joint infection (PJI) as end-point, survivorship was 95.7%. Three of the 47 patients (3/47 knees, 6.4%) required subsequent surgery. Two reoperations (4.3%) were for PJI developed 14 months and 3 years from index surgery. Both of them underwent 2-stage revision after antibiotic spacer placement. The duration between the stages was 6 weeks for both cases [29]. One of them was further complicated by a reinfection 8 months after revision TKA, and underwent a second two-stage revision and subsequent above-knee amputation. One patient underwent arthroscopic debridement for patella clunk syndrome. No further problems were encountered by the patient. One patient with a valgus knee (25°) developed a postoperative neuroapraxia of the peroneal nerve that was completely resolved in 6 months.

There was no evidence of loosening or migration of any implant at the latest follow-up. On the immediate postoperative AP radiographs in 5 knees (out of 47, 10.6%) there were radiolucent lines < 2 mm in thickness underneath the medial tibial tray at the bone–cement interface corresponding to zones 1 and 3 M on the AP view. Those lines were considered partial, stable, and asymptomatic at final follow-up. No radiolucent lines were reported underneath the lateral tibial tray (zone 2) or around the stem extensions, when those were used (zone 4). Those lines were considered nonprogressive and asymptomatic at latest follow-up. The mean standing postoperative femorotibial alignment was 3.7° of valgus (range between 1° of varus and 6° of valgus) and no patients had coronal deformity.

The mean Knee Society knee scores improved from 43 points (range 19–72 points) preoperatively to 86 points (range 54–100 points) postoperatively (*p* < 0.001). The mean Knee Society function scores improved from 40 points (range 17–69 points) preoperatively to 59 points (range 42–100 points) postoperatively (*p* < 0.001). Preoperatively, the average flexion contracture was 7° (range 0–15°) and the average flexion was 98° (range 75–110°). At the time of the latest follow-up, the average flexion contracture was 2° (range 0–5°) and the average flexion was 110° (range 90–120°) (Table 2). No knees reported varus–valgus instability in flexion or extension.

**Table 2:** Clinical outcomes of CCK implant in primary TKA

	Preoperative Mean (range)	Final follow-up Mean (range)	P value
KSKS	43 (19–17)	86 (54–100)	< 0.001
KSFS	40 (17–69)	59 (42–100)	< 0.001
Max flexion (°)	98° (75–105°)	108° (90–120°)	< 0.008
Flexion contracture	7° (0–15°)	2° (0–5°)	

*KSKS* Knee Society knee score,* KSFS* Knee Society function score

## Discussion

The findings of this study indicate that CCK implants in primary TKA with major coronal deformities and/or intraoperative inability to achieve an adequate stability with a standard PS implant, have a high overall survivorship (93.6%) at a mean follow-up of 9 years and survivorship from aseptic loosening of 100%, comparable with less constrained implants. These findings are in line with the recent systematic review and meta-analysis by Avino et al. [30], who reported significant clinical improvement in 3,620 CCK prostheses in primary TKA with a revision rate of 7% at 10-year follow-up.

Primary TKA in the setting of severe coronal deformity and/or intraoperative inability to balance the knee represents a unique challenge in knee reconstruction. Standard implants with a low level of constraint and adequate soft-tissue balancing should be considered the first choice in order to achieve a well-balanced and stable knee in primary TKA. When soft-tissue techniques are inadequate to obtain intraoperative stability throughout all ROM, increased constraint is necessary to avoid the risk of instability and subsequent early revision. In fact, instability represents one of the most frequent causes of early failure leading to revision TKA reaching up to 27% at 5 years [5]. Indications for a CCK implant have been previously described and include severe axial deformities, collateral ligament insufficiency, severe bone loss and persistent laxity > 7–10 mm [7–9, 31]. However, agreement over the exact amount of instability that requires increased constrained has not been reached yet. In addition, while providing an increased varus–valgus stability compared to CR and PS designs, CCK implants ensure an acceptable midterm survivorship, and represent a more reliable option than hinged designs in primary TKAs. According to Abdulkarim et al. [32], the overall survivorship of rotating hinge implants in complex primary non-oncologic TKA was 82% at a follow-up of 6–10 years.

Despite the disadvantages that have been associated with increased constraint, midterm survivorship from revision for aseptic loosening was 100%, which is in line with previous studies of primary CCK that reported no cases of failure of the implants for aseptic loosening at midterm follow-up in primary TKA [13, 14, 17, 19, 23, 31, 33]. Our results compare favorably with those of other studies [11, 15, 22, 34–37] (Table 3). No complications related to the tibial post mechanism were reported at a mean follow-up of 9 years (range 6–12 years). In these studies, a survivorship of CCK implants in primary TKA of > 95% at midterm follow-up was reported. In addition, no cases of periprosthetic fractures were reported in our series despite a reported incidence up to 3.2% in a cohort of 127 primary TKAs with CCK implants [33]. The transmission of the mechanical stress to the bone–cement interfaces may induce higher prevalence of loosening and subsequent osteolysis, especially when CCK implants are used in younger and high-demand patients. Johnson et al. [20], in their series of 21 knees in relatively young patients (mean 54 years), reported implant survivorship of 100% at 6-year follow-up and good functional outcomes despite a high incidence of stiffness (23.8%). Their results suggest that primary CCK may represent a valid option not only in elderly and low-demand patients, but also in younger and more active patients. Furthermore, according to Badawy et al. [38], based on the Norwegian Arthroplasty Register, complex primary TKAs that require an increased level of constraint have higher reoperation and revision rates at 2- and 5-year follow-up attributable to factors other than implant design. They found that revisions were most commonly due to infection although the midterm survivorship was similar to unconstrained implants. However, in their study, CCK implants had midterm aseptic survivorship comparable to unconstrained implants. Recently, mid-level constrained (MLC) articular bearing has been introduced as a promising alternative in cases of mild coronal deformity. It is characterized by a wider post that limits rotation and varus–valgus lift-off to a few degrees but is less constrained than a CCK insert. Crawford et al. [39], in a cohort of 103 stemless MLC articular-bearing implants, reported overall survivorship of 97% and survivorship from revision for aseptic loosening or instability of 100% at a mean follow-up of 5 years. Those findings are supported by Dubin et al. [40] in their series of 57 MLC knees compared with 96 PS knees with a mean follow-up of 4 years. The authors reported 3.5% (2 of 57) and 2% (2 of 96) revision rates, respectively, in patients with mild coronal deformity.

**Table 3:** Studies of CCK implants in primary total knee arthroplasty with minimum 5-year follow-up

Authors (year of publication)	No. of knees	No. of primary CCK	Mean age at index (years) (range)	Mean follow-up (years) (range)	Reoperations for aseptic loosening (rate)	Reoperations for infection (rate)	Reoperations for any reason (rate)	Infections (rate)
Lachiewicz et al. [12]	42	20	67 (40–91)	9 (5–16)	1 (5%)	0 (0%)	1 (5%)	0 (0%)
Lachiewicz et al. [13]	27	27	74 (28–94)	5 (2–12)	0 (0%)	0 (0%)	2 (7.4%)	2 (7.4%)
Nam et al. [34]	190	190	72	7	5 (2.6%)	1 (0.5%)	8 (4.2%)	1 (0.5%)
Maynard et al. [33]	127	107	68 (42–86)	9 (7–13)	0 (0%)	2 (1.6%)	13 (10.2%)	4 (3.1%)
Cholewinski et al. [18]	43	43	66 (21–88)	12 (10–14)	0 (0%)	2 (4.7%)	8 (18.6%)	4 (9.3%)
Luque et al. [11]	89	89	71	7 (2–8)	2 (2.2%)	6 (6.7%)	11 (12%)	6 (6.7%)
Ruel et al. [35]	142	142	N/A	5 (2–10)	7 (4.9%)	2 (1.4%)	14 (9.9%)	2 (1.4%)
Martin et al. [22]	427	427	65	5 (2–12)	19 (4.4%)	5 (1.2%)	75 (17.6%)	26 (6.1%)
Feng et al. [19]	48	15	61 (29–81)	6 (3–8)	0 (0%)	0 (0%)	0 (0%)	0 (0%)
Ye et al. [17]	51	31	64 (52–76)	6 (4–8)	0 (0%)	1 (3.2%)	1 (2%)	1 (3.2%)
Tripathi et al. [37]	100	100	59 (45–70)	7	1 (1%)	1(1%)	3 (3%)	2 (2%)
Siqueira et al. [37]	247	247	67	8 (2–15)	2 (0.8%)	13 (5.3%)	22 (8.9%)	13 (5.3%)
Li et al. [14]	43	43	65 (60–72)	5 (2–10)	0 (0%)	0 (0%)	2 (4.7%)	0 (0%)
Johnson et al. [20]	21	21	54 (39–59)	6 (2–12)	0 (0%)	0 (0%)	2 (9.5%)	0 (0%)
Mancino et al. (current study)	47	47	72 (43–86)	9 (6–12)	0 (0%)	2 (4.3%)	3 (6.4%)	2 (4.3%)

N/A not available

Our overall complication rate was 8.5% (4 out of 47) and reoperation rate was 6.4% (3 out of 47). Infection was the most frequent complication and the most common reason for reoperation (2 of 47, 4.3%). According to Jamsen et al. [41], in a register-based analysis, there was a trend showing an increased rate of infections in association with constrained and hinged prostheses at 3 years (1.2%) compared with non-constrained CR or PS implants (0.7%); this trend was significant especially for primary osteoarthritis. Our incidence of implant revision for PJI was 4.3%, supporting the findings of a previous series where the most common reason for revision TKA was infection. Cholewinski et al. [18] and Luque et al. [11], reported revision rates for infection of 4.7% (2 of 43 knees) and 6.7% (6 of 99 knees) in their series of 43 and 99 knees at a mean follow-up of 12 and 7 years, respectively. Our results were higher than other studies of CCK implants in primary CCK reported in the current literature [12–14, 17, 19, 20, 23, 33] (Table 3). Interestingly, a recent retrospective analysis of > 18,000 primary TKAs, reported a higher overall failure rate in patients with a primary CCK implant compared to a standard PS implant, with a hazard ratio of 1.99. This finding may be related to the fact that patients with CCK implants may present poor bone stock, ligamentous deficiency, or large preoperative deformities [42]. In addition, Costa et al. [43] compared the outcomes of 74 primary CCK implants with 73 primary PS implants. The authors reported a higher risk of early PJI in patients who underwent primary CCK compared to PS TKA, confirming that protracted surgical time attributed to greater difficulty in exposure and in achieving adequate balance, representing a predisposing factor for PJI.

Radiolucent lines were found in 10.6% of the cases. All of them were < 2 mm in thickness and found underneath the medial tibial tray at the bone–cement interface corresponding to zones 1 and 3 M on the AP view according to Meneghini et al. [28]. Those lines were considered partial and stable according to the same criteria, and asymptomatic at final follow-up. No radiolucent lines were reported underneath the lateral tibial tray (zone 2) or around the stem extensions when those were used (zone 4). In addition, no radiolucent lines were reported around the femoral component. These findings were in line with those reported by Maynard et al. (9.4%) [33] and Lachiewicz et al. (11%) [13]. The authors reported an incidence of radiolucent lines of 9.4% (14 of 127) and 11% (3 of 27) in their series of 127 and 27 knees at a mean follow-up of 9 and 5 years, respectively. An incidence of non-progressive radiolucent lines underneath the tibial component in primary CCK has been reported in up to 47% of cases [12, 18], likely suggesting incomplete cement pressurization.

The Knee Society knee score improved by an of average 43 points from the preoperative state to the latest follow-up, and the functional score improved by a mean 19 points. The improvement between mean pre- and postoperative values was comparable with what has been previously reported in other studies of primary CCK with significant improvement in clinical outcomes and flexion ROM [11–13, 17, 18, 20, 23]. The ROM improved by mean 10° of flexion, in line with what is reported in the literature [12, 17, 20, 23, 33], and higher than reported by Feng et al. [19]. Flexion contracture decreased from mean 7° in the preoperative evaluation to mean 2° at final follow-up. Puah et al. [15] recently reported no significant difference in ROM or functional scores between primary CCK implants and PS implants at 6 months and 2 years.

There are some limitations to this study. First, the current study is a retrospective study with all the limitations of this type of design. Second, the patient cohort was relatively small and lacked a control group matched for baseline characteristics with lower constraint implants. Third, some selection bias might have been detected since intraoperative indication to a CCK implant was left to the surgeon’s preference and experience. Fourth, the implants were used in relatively elderly patients (mean age 72 years) with relatively low demands; however, the results may differ in younger, more active and demanding patients.

## Conclusion

CCK implants in primary TKA can be considered a viable option in cases of severe coronal deformities or when intraoperative stability is not achievable with standard implants (CR, PS, MS), providing good survivorship and functional outcomes at midterm follow-up, comparable with less constrained implants. With the recent introduction of mid-level constrained implants the need for CCK implants in primary TKA may decrease. However, a higher degree of constraint should be used cautiously and patients should be carefully selected, leaving the first choice to implants with a lower level of constraint since increased mechanical stresses may affect long-term survivorship.

## Data Availability

The datasets generated and/or analysed during the current study are not publicly available due to the high volume of data but are available from the corresponding author on reasonable request. This work was performed at the Adult Reconstruction and Joint Replacement Service, Division of Orthopaedics and Traumatology, Department of Aging, Neurological, Orthopaedic and Head-Neck studies, Fondazione Policlinico Universitario Agostino Gemelli IRCCS, Roma, Italy

## Abbreviations

TKA: Total knee arthroplasty
CR: Cruciate-retaining
PS: Posterior-stabilized
MS: Medial-stabilized
CCK: Condylar-constrained knee
ROM: Range of movement
AORI: Anderson Orthopaedic Research Institute
KSS: Knee Society score
